# Preventive Effect of Muscone against Cisplatin Nephrotoxicity in LLC-PK1 Cells

**DOI:** 10.3390/biom10101444

**Published:** 2020-10-15

**Authors:** Hung Manh Phung, Sullim Lee, Ji Hye Hwang, Ki Sung Kang

**Affiliations:** 1 College of Korean Medicine, Gachon University, Seongnam 13120, Korea; manhspkt92@gmail.com; 2 Department of Life Science, College of Bio-Nano Technology, Gachon University, Seongnam 13120, Korea; sullimlee@gachon.ac.kr; 3 Department of Acupuncture and Moxibustion Medicine, College of Korean Medicine, Gachon University, Seongnam 13120, Korea

**Keywords:** muscone, cisplatin, nephrotoxicity, acute kidney injury, LLC-PK1, chromatin condensation, apoptosis, oxidative stress, inflammation, p53

## Abstract

Cisplatin, one of the most common antitumor agents, is widely applied to treat various cancerous diseases and is included in the World Health Organization Model List of Essential Medicines. Cisplatin therapy is used to treat 10–20% of all cancerous cases, and its cure rate is especially high in testicular cancer (over 90%). However, a major side effect of this anticancer drug is nephrotoxicity, limiting treatment effect and reducing the quality of life in cancer patients. Muscone, an odoriferous constituent of musk, was confirmed to inhibit cisplatin-induced LLC-PK1 kidney proximal tubule cell death in a dose-dependent manner. In term of renal protective mechanism, muscone inhibited cisplatin oxidative toxicity by decreasing reactive oxygen species (ROS) level and stimulating HO-1 expression. Muscone also exerted anti-inflammation effect through inhibition of p38 phosphorylation. Furthermore, muscone mitigated cisplatin-induced apoptosis in LLC-PK1 cells via both intrinsic and extrinsic pathways by inhibiting pro-apoptotic protein Bax expression, and cleaved caspase-3, 7, and 8; and increase of anti-apoptotic protein Bcl-2 level. In addition, the anti-apoptotic effect of muscone also was enhanced by preventing p53 expression and its phosphorylation. Our study showed that muscone may be a potential protective agent against cisplatin-induced nephrotoxicity.

## 1. Introduction

Cisplatin, known as *cis*-diamminedichloroplatinum, is one of the most common antineoplastic agents used to treat various cancerous diseases, including ovarian, breast, testicular, brain, lung, and bladder cancer, and is included in the World Health Organization Model List of Essential Medicines. Cisplatin inhibits carcinogenesis of malignant cells through the generation of intra- and interstrand cross-links on DNA, which block DNA synthesis and replication [1,2,3,4]. The curing rate of cisplatin is particularly high in testicular cancer (over 90%) [5] and 10–20% of all cancerous cases are prescribed cisplatin-based chemotherapy [6]. 

Although cisplatin is considered a cornerstone of cancer therapy, its clinical effectiveness is limited owing to adverse reactions in normal tissues. As a primary organ involved in the excretion of cisplatin, the accumulation of cisplatin in the kidney is greater than that in other organs [7]. Thus, renal toxicity is considered one of the biggest challenges in cisplatin-based chemotherapy besides other common side effects such as gastrointestinal toxicity, ototoxicity, neurotoxicity, and hematological toxicity. Approximately one-third of patients undergoing cisplatin treatment show symptoms of acute kidney injury (AKI), including higher serum creatinine, lower glomerular filtration rate, and decreased serum potassium and magnesium concentrations after 10-day drug administration [8]. 

The nephrotoxic mechanism of cisplatin remains unknown despite decades of research. Recent studies have indicated that cisplatin induces renal failure by triggering complex intracellular signaling pathways. In brief, after cisplatin is transported through high-affinity copper uptake protein 1 (CTR1), organic cation transporters (OCTs), or passive diffusion via the cell membrane, it triggers a series of signaling pathways including those affecting the balance of cdk2 and p21, activation of mitogen-activated protein kinase (MAPK), tumor necrosis factor-α, tumor protein p53, nuclear factor-κB, mitochondrial dysfunction, DNA damage, reactive oxygen species (ROS) generation, and formation of toxic metabolites. These upstream changes result in apoptosis or inflammation downstream, which leads to renal cell death and AKI [8].

Although cisplatin therapy presents risks for kidneys and other organs, the clinical effectiveness of cisplatin is undeniable in cancer treatment. For years, various solutions have been proposed to reduce nephrotoxicity and conserve the therapeutic effect of cisplatin, such as the synthesis of new cisplatin-like drugs that express low toxicity on normal tissues (carboplatin) [9] or hydration of the patients during cisplatin administration [10]. In addition, in recent years, the combination of cisplatin therapy with renal protective supplements originating from natural sources has been widely researched in both in vitro and in vivo models [11,12,13,14,15,16].

**Figure 1:**
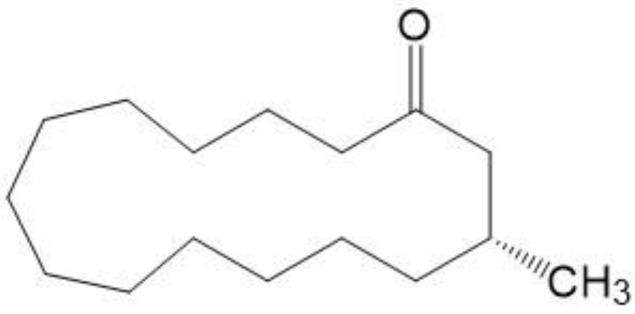
Chemical structure of muscone.

Musk, well known as a high-value crude drug in traditional Chinese medicine, is produced from ventral glandular secretion of the male musk deer, and is widely applied in pain relief, promotion of blood flow, and resuscitation. In addition, muscone (3-methylcyclopentadecanone, Figure 1), the key compound regulating the odor of musk, also possesses several valuable pharmaceutical activities such as anti-early pregnancy, anti-cerebral ischemia, anti-cancer, neuroprotective, and cardioprotective effects [17]. Previous reports have shown that muscone has a protective effect on various normal cell lines, including neurons and cardiac myocytes. The common feature in the cellular protective mechanisms of muscone on these cell types is the inhibitory effect on diverse cell death pathways such as oxidative stress, inflammation, and apoptosis, which share the pathway of cisplatin-induced nephrotoxicity [8,18,19]. Furthermore, Liu et al. [20] indicated that muscone strengthened the treatment effect of bone marrow stromal cells (BMSCs) therapy in AKI by stimulating the migration, survivability, and proliferation of BMSCs and inhibiting the expression of inflammatory cytokines and apoptosis in renal tissues. Based on these studies, muscone may have a preventive effect against cisplatin-induced kidney cell death. In this study, we assessed the cytoprotective effect of muscone against cisplatin nephrotoxicity in LLC-PK1 kidney proximal tubule cells. 

## 2. Materials and Methods 

### 2.1. Cell Culture and Drug Treatment

LLC-PK1 cells (ATCC, Manassas, VA, USA) were propagated in Medium 199 (Welgene, Gyeongsangbuk, Korea) containing 10% fetal bovine serum (Regeneration Biology, Ottawa, Canada) and 1% streptomycin-penicillin (Corning, Manassas, VA, USA). The typical growth conditions were set as 37 °C, 95% relative humidity, and 5% CO_2_. A 100 mM stock solution of muscone (The Nature Network, Vestenbergsgreuth, Germany) and N-acetylcysteine (NAC; Abcam, Cambridge, UK) were prepared in dimethyl sulfoxide (DMSO; Santa Cruz Biotechnology, TX, USA). A 2 mM stock solution of cisplatin (Sigma-Aldrich, St. Louis, MO, USA) was prepared in autoclaved distilled water. The final proportion of DMSO was controlled to 0.1%, at which concentration cytotoxicity of vehicle (DMSO) and inhibition of cisplatin could not be observed in comparison to that in the non-treated cells. For experiments, LLC-PK1 cells were seeded into multi-well plates at a density of 3 × 10^4^ cells/cm^2^ and incubated for 24 h. Next, the cells were treated with specific doses of compounds for 2 h and subsequently 20 µM cisplatin.

### 2.2. Assessment of Cell Viability 

LLC-PK1 cells were treated with 12.5, 25, 50 and 100 µM muscone, 100 and 500 µM NAC (reference drug) in the absence or presence of 20 µM cisplatin in a 96 well-plate for 24 h. The medium in each well was replaced with 10% EZ-Cytox (Dogen, Seoul, Korea) solution. Subsequently, the plate was kept in an incubator for 30 min and the OD_450_ was measured using a microplate reader (SPARK 10M; Tecan, Männedorf, Switzerland). The viability of cells was calculated as a percentage relative to the viability of control cells.

### 2.3. Intracellular ROS Assay 

After 24 h seeding onto a 6 well-plate, the cells were exposed to 50 and 100 µM muscone for 2 h and continuously treated or nontreated with 20 µM cisplatin for 24 h. Subsequently, the cells were stained with 10 µM 2′, 7′-dichlorofluorescein diacetate (DCFDA; Thermo Fisher Scientific, Waltham, MA, USA) for 30 min and then washed with PBS. The images were obtained using an IX51 fluorescence microscope (Olympus, Tokyo, Japan) connected to a CCD camera and analyzed using an ImageJ software (Version 1.51 J, National Institutes of Health, Bethesda, MD, USA).

### 2.4. Determination of Nuclear Condensation

Chromatin condensation was assessed according to the protocol described by Xia et al. (2019) with some modifications [21]. After 8 h treatment with 50 and 100 µM muscone in the absence or presence of 20 µM cisplatin, the LLC-PK1 cells were exposed to 10 µM Hoechst 33258 (R&D Systems, Minneapolis, MN, USA) and protected from light for 10 min. The remaining dye was removed using phosphate-buffered saline (PBS; Welgene, Gyeongsangbuk, Korea), and the nuclei of cells were imaged using an IX51 fluorescence microscope (Olympus, Tokyo, Japan) connected to a CCD camera. 

### 2.5. Tali^®^ Assay

The number of apoptotic cells was determined using the Tali^®^ Apoptosis Kit (Thermo Fisher Scientific, Eugene, OR, USA). After exposure to 50 and 100 µM muscone in the presence or absence of 20 µM cisplatin for 8 h, the cells were harvested, washed with PBS, stained with annexin V, and protected from light. After 20 min, the cells were continuously washed with PBS, and then stained with Annexin V and propidium iodide to detect apoptotic and dead cells. A Tali^®^ Image-Based Cytometer (Invitrogen, Carlsbad, CA, USA) was used to capture fluorescence images. The proportion of apoptotic cells was computed using TaliPCApp, version 1.0. 

### 2.6. Immunoblotting Analysis

Western blotting analysis was performed according to the procedure described by Jang et al. (2018), with a few modifications [22]. After treatment for 24 h with 50 and 100 µM muscone in the absence or presence of 20 µM cisplatin in a 6 well-plate, LLC-PK1 cells were washed with PBS and lysed in 1× RIPA buffer (Cell Signaling, Danvers, MA, USA) supplemented with 1 mM phenylmethylsulfonyl fluoride (Abcam, Cambridge, UK). The whole cell lysate was centrifuged at 16,000× *g* for 20 min at 4 °C to collect the supernatant. The BCA Protein Assay Kit (Abcam, Cambridge, UK) was used to measure the concentration of each protein sample. Equal amounts of protein (10 µg/lane) were separated on a polyacrylamide gel, transferred to a polyvinylidene difluoride membrane (Sigma-Aldrich, St. Louis, MO, USA) presoaked in methyl alcohol, and blocked in 5% nonfat milk in tris-buffered saline supplemented with 0.1% Tween 20 (TBS-T) for 1 h. Subsequently, the membranes were probed with the first antibodies of interest overnight at 4 °C and secondary antibodies for 1 h. All antibodies were supplied by Cell Signaling (Danvers, MA, USA). Next, the membranes were exposed to a mixture of reagents A and B at a 1:1 ratio (ECL Western Blotting Substrate Kit, Abcam, Cambridge, UK), and the immunoreactive bands were developed using the Fusion Solo Chemiluminescence System (PEQLAB Biotechnologie GmbH, Erlangen, Germany). 

### 2.7. Statistical Methods

The experimental results were described as the mean ± standard deviation (SD). Statistical differences were assessed using one-way analysis of variance (ANOVA) with the Tukey’s range test. Statistical significance was set at *p*-values of less than 0.05. The IBM^®^ SPSS^®^ Statistics software (Version 25, IBM, Armonk, NY, USA) was used to conduct the statistical evaluation.

## 3. Results and Discussion

**Figure 2:**
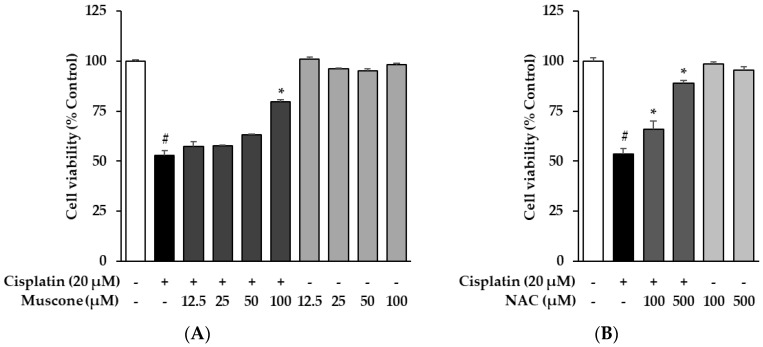
Protective effects of muscone against cisplatin-induced cytotoxicity in kidney proximal tubule LLC-PK1 cells. (**A**) LLC-PK1 cells were treated with 20 µM cisplatin and indicated doses of muscone for 24 h; cell survival was detected using the Ez-Cytox cell viability assay kit. (**B**) NAC was used as the reference drug. The results are described as the mean ± SD (*N* = 3). Notes: # *p* < 0.05 compared with the non-treated group, * *p* < 0.05 compared with the cisplatin treated group.

The protective effect of muscone against cisplatin-induced kidney proximal tubule LLC-PK1 cell death was examined using cell viability assay. The NAC, a medication primarily is used as a mucolytic agent and in treatment of paracetamol overdose proved the effectiveness in reduction of cisplatin nephrotoxicity in both in vitro and in animal models, which was chosen as the reference drug [23,24]. As indicated in Figure 2, the viability of cells reduced to 53.3 ± 2.3% (*N* = 3, *p* = 0.05) in cisplatin only-treated cells and was restored in a concentration-dependent manner in the presence of compounds. In detail, the muscone possessed the nephroprotective effect stronger than NAC compared at the same dose. The cells treated with 100 µM muscone recovered viability of LLC-PK1 cells to 79.9 ± 1.0% (*N* = 3, *p* = 0.05) while exposure to 100 µM NAC only increased cell viability to 65.9 ± 4.2% (*N* = 3, *p* = 0.05). NAC required 500 µM to recover the viability of cell to 88.8 ± 1.5%. These results indicated that the potential of muscone in reversing the nephrotoxicity of cisplatin. Subsequently, mechanistic assays were performed to clarify how this compound expressed its nephroprotective effects.

The oxidative stress is widely known as an important factor promoting tubule damage caused by cisplatin. When cisplatin enters the cells, it stimulates ROS generation via glutathione inactivation, mitochondrial dysfunction and cytochrome P450 (CYP) system. The accumulation of ROS not only triggers various forms of cellular damage, consisting of lipid peroxidation, DNA damage, and protein denaturation but also involves in early state of various signaling pathways, which induce renal cell death and kidney failure. Beside renal damage, oxidative stress also activates antioxidant defense system including heme oxygenase 1 (HO-1). The previous studies pointed out that overexpression of HO-1 inhibited cisplatin- induced apoptosis in human renal proximal tubule cells and prevented AKI in rats [8]. 

Regarding to muscone, its antioxidant activity has been mentioned in various reports [17,18,25]. For instance, Du et al. [25] showed that muscone attenuated lipopolysaccharide-induced ROS formation in bone marrow-derived macrophages. The inhibitory effect of muscone on ROS generation also was observed in PC12 cells caused by glutamate [18]. Hence, we stained LLC-PK1 cells with DCFDA accompany by evaluation of HO-1 expression using Western blotting analysis to clarify preventive effect of muscone against cisplatin oxidative toxicity.

**Figure 3:**
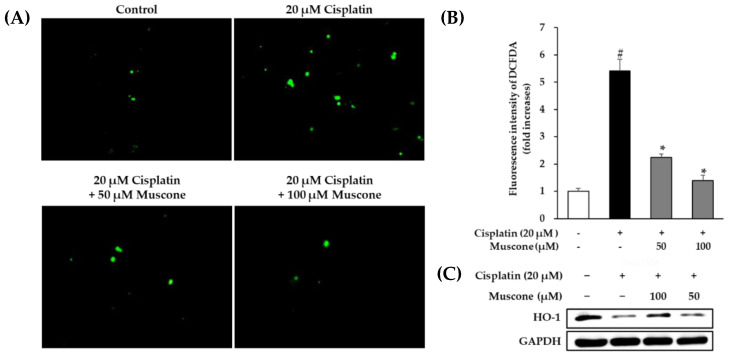
Preventive effect of muscone against cisplatin induced oxidative stress in kidney proximal tubule LLC-PK1 cells. (**A**) After 24 h treatment with specific doses of muscone in the presence or absence of cisplatin, LLC-PK1 cells were stained with 10 µM DCFDA for 30 min. The images were obtained using an IX51 fluorescence microscope (Olympus, Tokyo, Japan) connected to a CCD camera. (**B**) The bar chart describes the fold increases in DCFDA fluorescence intensity analyzed from images using ImageJ software. (**C**) The effect of muscone on expression of HO-1 after 24 h exposure to indicated concentration of muscone and 20 µM cisplatin in LLC-PK1 cells. The data are described as the mean ± SD (*N* = 2). Notes: # *p* < 0.05 compared with the control group, * *p* < 0.05 compared with the cisplatin treated group.

It is clear in Figure 3A that muscone decreased level of cisplatin-induced ROS in a concentration- dependent manner. Furthermore, the qualitative dada analyzed from fluorescence images indicated that treatment with 20 µM cisplatin increased fluorescence intensity of DCFDA to 5.4 ± 0.4% fold (*N* = 3, *p* = 0.05) and decreased to 2.2 ± 0.1% fold (*N* = 3, *p* = 0.05) and 1.4 ± 0.2% fold (*N* = 3, *p* = 0.05) in group treated with 50 and 100 µM muscone, respectively (Figure 3B). In addition, exposure to muscone activated cytoprotective response against cisplatin nephrotoxicity via increase of HO-1 expression (Figure 3C). These data showed that muscone exerted an antioxidant effect through neutralization of cisplatin oxidative toxicity.

**Figure 4:**
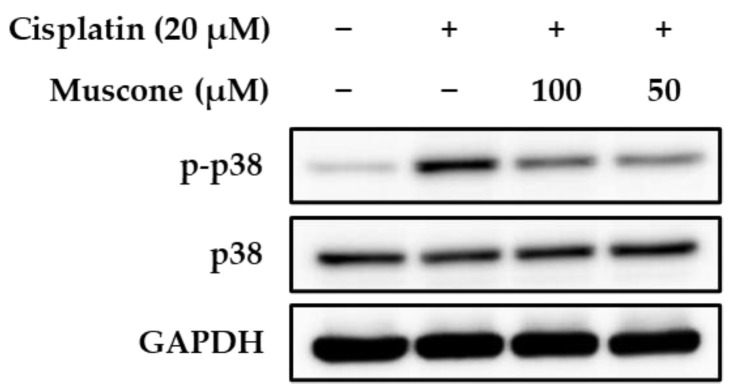
Effect of muscone on phosphorylation of p38 in kidney proximal tubule LLC-PK1 cells. The cells were exposed to indicated doses of muscone in the presence or absence of cisplatin for 24 h. Protein expression was assessed using immunoblotting.

Cisplatin-induced ROS generation triggers several renal cell death pathways including p38 signaling pathway. Treatment with SB203580 and SKF-860002 as the p38 inhibitors reversed renal toxicity of cisplatin in both in vitro and in vivo models. In addition, the p38 protein does not affect directly to renal cell death and injury, it may mediate production of TNF-α cytokine in renal cell, which results in inflammation response during cisplatin nephrotoxicity [8,26]. Thus, we conducted the immunoblotting analysis to examine the effect of muscone on phosphorylation of p38 induced by cisplatin in LLC-PK1 cells. As shown in Figure 4, muscone inhibited cisplatin-induced phosphorylated p38, which may a potential indicator for anti-inflammatory effect of muscone against cisplatin toxicity. 

In terms of pathology, renal vascular dysfunction, inflammation, and tubular damage are known as the typical hallmarks of AKI. It is generally held that the death and injury of tubular cells play a key role in AKI progression. While sublethal injury can be recovered, tubular cell death tends to induce cellular dysfunction, activation of inflammatory factors, and damage-associated molecular patterns in tissue injury. In AKI, apoptosis and necrosis are considered primary pathway-induced renal cell death processes [27]. Previous reports indicated that a high concentration of cisplatin induces necrosis, whereas apoptotic tubular cells are detected mainly at lower doses of cisplatin treatment [8]. In this study, we mainly focused on the apoptosis mechanism to investigate the renal protective effect of muscone.

**Figure 5:**
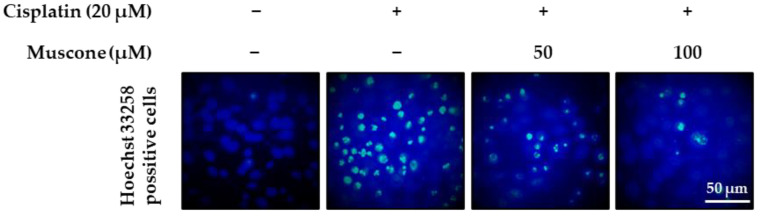
Protective effect of muscone against cisplatin-induced chromatin condensation in kidney proximal tubule LLC-PK1 cells. LLC-PK1 cells were exposed to muscone in the presence or absence of 20 µM cisplatin for 8 h and dyed with Hoechst 33258. Fluorescent images were captured using a fluorescent microscope (scale bar: 50 µm).

In the early stage, apoptotic cells are characterized by various morphological changes, including pyknosis, cell shrinkage, and plasma membrane blebbing. One of the most typical characteristics of cells undergoing apoptosis is chromatin condensation, which distinguishes it from necrosis [28]. Hence, we first examined the anti-apoptotic effect of muscone via observation of nuclear condensation using Hoechst 33258 staining. As shown in Figure 5, exposure to 20 µM cisplatin increased chromatin condensation in LLC-PK1 cells, whereas treatment with muscone reduced it in a dose-dependent manner. 

**Figure 6:**
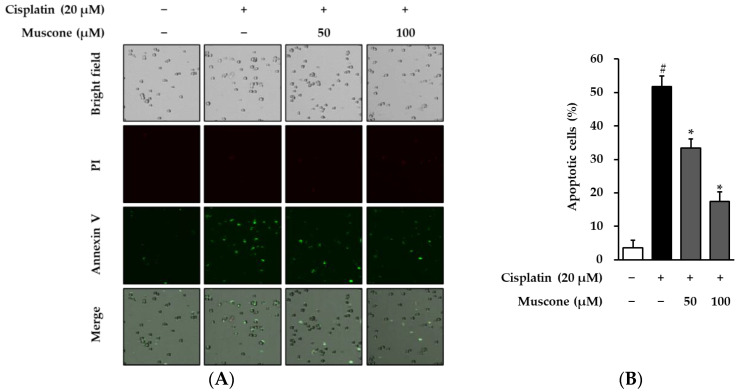
Protective effect of muscone against cisplatin induced apoptosis in kidney proximal tubule LLC-PK1 cells. (**A**) LLC-PK1 cells were exposed to indicated concentrations of muscone in the presence or absence of 20 µM cisplatin for 8 h and dyed with propidium iodide (PI) and Alexa Fluor 488-conjugated annexin V. (**B**) The bar chart depicts the percentage of apoptotic cell (annexin V-positive cells) quantified from images obtained using TaliPCApp. The data are described as the mean ± SD (*N* = 2). Notes: # *p* < 0.05 compared with the non-treated group, * *p* < 0.05 compared with the cisplatin treated group.

To confirm the preventive effect of muscone against cisplatin-induced apoptosis in LLC-PK1 cells, propidium iodide and annexin V dual staining was applied to quantify necrotic and apoptotic cells. From Figure 6A, we can see that muscone reduced the amount of apoptotic cells (annexin V-positive cells) in a concentration-dependent manner compared to cisplatin only-treated cells. In addition, the quantitative results indicated that treatment with 20 µM cisplatin increased the proportion of apoptotic cells to 51.7 ± 3.2%, which decreased to 33.4 ± 2.7% and 17.5 ± 2.8% in the presence of 50 µM and 100 µM muscone, respectively (Figure 6B). Based on Hoechst staining and Tali^Ⓡ^ assay data, muscone initially expressed the effect in protecting LLC-PK1 cells against cisplatin-induced apoptosis. However, to clarify how muscone inhibited the apoptosis signal caused by cisplatin, we assessed its effect on the expression of proteins-regulated programmed cell death processes.

Once cisplatin enters the renal cells through CTR1, OCTs, or passive diffusion via the cell membrane, it activates several apoptotic pathways, including the endoplasmic reticulum stress (ER) pathway, an intrinsic pathway centered on mitochondria and an extrinsic pathway triggered via death receptors. It is generally believed that the intrinsic or mitochondrial pathway is the primary apoptotic pathway in the renal toxicity of cisplatin. Treatment of kidney epithelial cells with cisplatin stimulates translocation of the pro-apoptotic protein Bax from the cytosol to the mitochondrial membrane, resulting in the release of apoptosis-inducing factor (AIF) and cytochrome *c.* The leaked cytochrome *c* binds to apoptosis activating factor 1 proteins to form an apoptosome, causing activation of caspase 9 and downstream caspases for caspase-dependent apoptosis (caspase-3, 7), whereas the release of AIF into the cytosol and its translocation from the cytoplasm to the nucleus causes caspase-independent apoptosis. With regard to the extrinsic pathway, previous reports have shown that cisplatin may activate death receptors comprising tumor necrosis factor receptors (TNFRs) and Fas, which leads to the activation of caspase-8 regulating downstream caspase-induced cell death. Cisplatin nephrotoxicity is also involved in ER stress-induced activation of Ca^2+^-dependent phospholipase A_2_ and caspase-12. In addition, cisplatin-induced DNA damage triggers p53 leading to induct apoptosis genes consisting of p53-induced protein with death domain (PIDD) and p53–upregulated modulator of apoptosis (PUMA-α). Activation of these genes induces release of AIF and cytochrome *c* from mitochondria through interaction with caspase 2 and Bcl-2 family proteins (Bcl-XL and Bax), which causes apoptosis [8,26].

**Figure 7:**
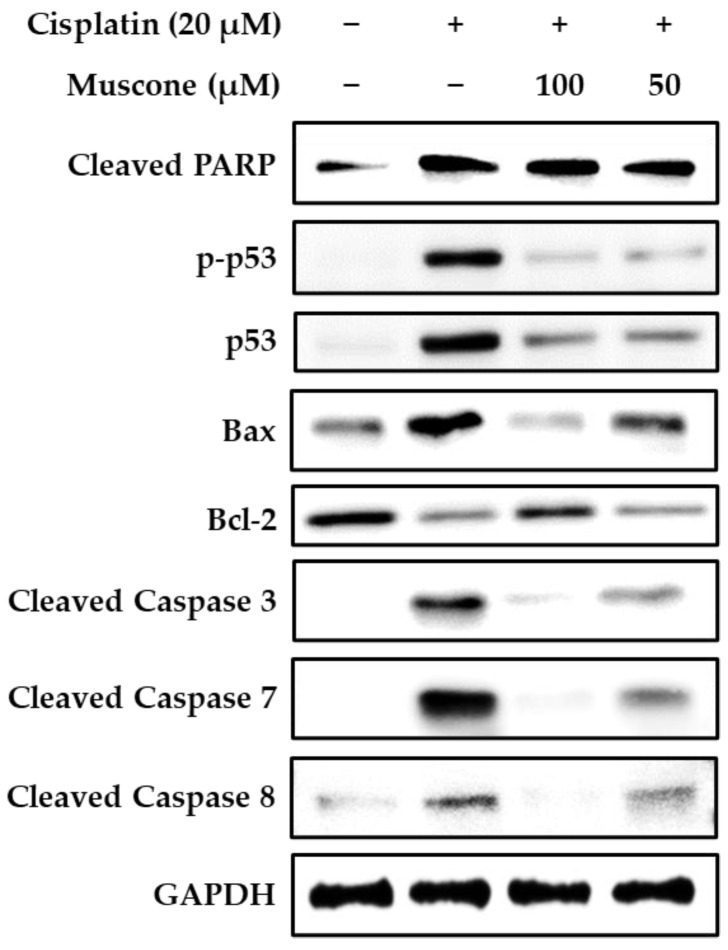
Effect of muscone on the expression of proteins-regulated in kidney proximal tubule LLC-PK1 cells. The cells were treated with 20 µM cisplatin and specific concentration of muscone for 24 h. Protein expression was determined by western blotting.

In our present study, muscone reduced both the expression and the phosphorylation of p53. Besides, it also inhibited the expression of pro-apoptotic protein Bax, cleaved caspase-3, 7, and 8; and recovered the level of anti-apoptotic protein Bcl-2; however, muscone failed to reduce cleaved poly (ADP-ribose) polymerase (PARP) level, which is an indicator of DNA damage (Figure 7). These data indicated that muscone attenuated cisplatin-induced apoptotic LLC-PK1 cell death via both intrinsic and extrinsic pathways by inhibiting the cleavage of caspase-3, 7, and 8, and the protein Bax expression; and increasing the level of protein Bcl-2. In addition, muscone also reversed p53 pathway by preventing cisplatin-induced p53 expression and phosphorylation, which protects kidney proximal tubule LLC-PK1 cells against apoptosis.

**Figure 8:**
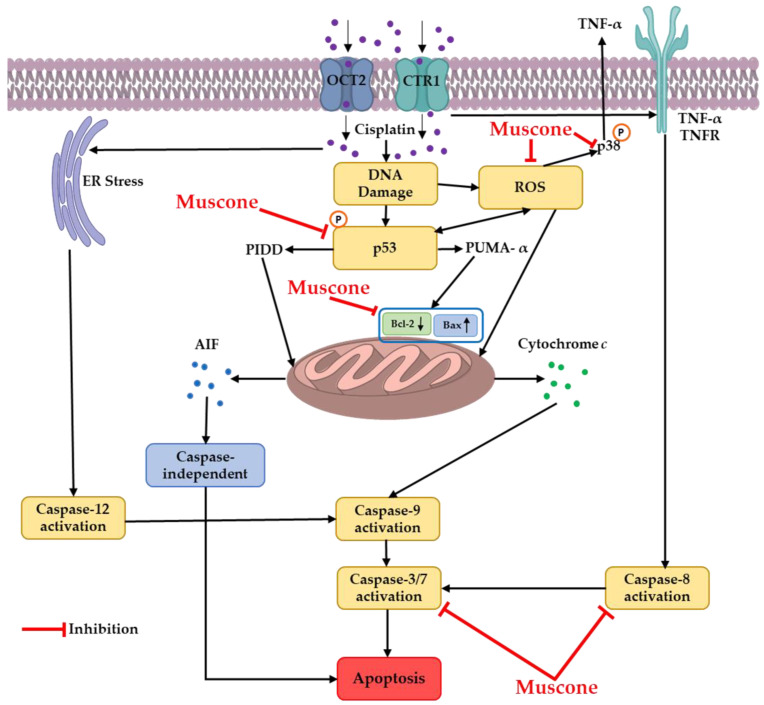
Cytoprotective mechanism of muscone against cisplatin nephrotoxicity in kidney proximal tubule LLC-PK1 cells.

The cytoprotective mechanism of muscone targeted on 3 main pathways including oxidative stress [29], inflammation and apoptosis [16]. In previous our reports under the same conditions, we mainly assessed MAPK, cleaved caspase 3 and p53 to evaluate protective mechanism of compound against cisplatin nephrotoxicity on LLC-PK1 cells. For instances, monoacetate from *Poria cocos* Wolf inhibited oxidative stress-regulated apoptosis via reducing MAPK phosphorylation and cleaved caspase 3 [30]. Ginsenosides Rg3, Rg5, and Rk1 found in processed ginseng prevented LLC-PK1 cells against cisplatin-induced apoptosis and inflammation by inhibition of c-Jun N-terminal kinase (JNK) phosphorylation, p53 expression and cleavage of caspase 3 [31]. In this study, the renal protective mechanism of muscone against cisplatin toxicity in LLC-PK1 cells was focused on interaction among p53, ROS, p38 and apoptosis pathways as shown in Figure 8. The inhibitory effect of muscone on ROS generation and p53 expression and phosphorylation may inhibit intrinsic pathway of apoptosis and p38 pathway inducing inflammation cytokine (TNFα).

In summary, muscone protected LLC-PK1 kidney proximal tubule cells against cisplatin nephrotoxicity in a concentration-dependent manner. Muscone expressed the inhibitory effect against cisplatin-induced oxidative stress by decrease of ROS accumulation and increase of HO-1 expression. Besides, muscone also displayed the anti-inflammatory activity via inhibiting the phosphorylation of p38. Furthermore, muscone prevented LLC-PK1 cells from cisplatin-induced apoptosis by decreasing chromatin condensation, the expression of the pro-apoptotic protein Bax and cleavage of caspase-3, 7, and 8; and increasing level of anti-apoptotic protein Bcl-2. In addition, the anti-apoptotic effect of muscone also was enhanced by preventing p53 expression and phosphorylation. The protective mechanism of muscone against cisplatin nephrotoxicity in LLC-PK1 cells is summarized in Figure 8. 

However, we are aware that our research may exist some limitations compared to previous reports at the same scope. Firstly, the effect of muscone on other cell death pathways caused by cisplatin including autophagy [32] and nuclear factor-κB activation [33] has not been investigated yet in this study. Secondly, the experimental results are just at in vitro scope. This work should be further confirmed at in vivo level to clarify the protective effect of muscone against cisplatin nephrotoxicity in living organisms [34].

## 4. Conclusions

Our experimental results showed that muscone prevented cisplatin-induced oxidative stress, inflammation and apoptosis in LLC-PK1 cells. Muscone was proved as a promising antioxidant via inhibiting ROS accumulation caused by cisplatin and stimulating HO-1 expression. Muscone also expressed anti-inflammation activity by attenuation of phosphorylated p38. In addition, the effect of muscone against cisplatin-induced apoptotic LLC-PK1 cell death was mediated through inhibition of p53, caspase-3, 7, and 8, and level of bax, and recover of Bcl-2 expression. Thus, muscone may be a potential protective agent against the renal toxicity of cisplatin.
